# Introduction

**DOI:** 10.1155/S1070360894000123

**Published:** 1994

**Authors:** Harubumi Kato


					Diagnostic and Therapeutic Endoscopy, Vol. 1, p. v
Reprints available directly from the publisher
Photocopying permitted by license only
(C) 1994 Harwood Academic Publishers GmbH
Printed in Malaysia
Introduction
Medical instrumentation has developed remarkably in the past several decades. One area in which progress has been par
ticularly noticeable is endoscopy. The development of fiberoptic endoscopes in the late 1950's and 1960's opened a new
era in terms of diagnostic procedures, including simpler anesthesia techniques and reduced discomfort for the patient.
With the advent of the 1970's and 1980's endoscopy came to be increasingly used not only for diagnostic but also for
therapeutic procedures. In particular, the application ofdifferent kinds oflasers in combination with endoscopes increased
the diagnostic and therapeutic value of these instruments. More recently the advent of electronic videoendoscope has in-
creased the possibilities of diagnostic applications as well as information storage and transfer.
Diagnostic and Therapeutic Endoscopy aims to publish materials on all topics related to endoscopic procedures in all
organs. As a result, we encompass ENT, the tracheobronchial tree, esophagus, upper GI, hepatopancreto-biliary system,
lower GI, urinary bladder, female reproductive tract, joints, and vascular, thoracic and abdominal cavities. The journal
will be published quarterly and will include full-length papers, review articles and case reports. Our aim is to ensure not
only a high scientific level but also an excellent level ofphotography, since it is the quality of photographs that determine
the value of papers or endoscopic findings. Articles concerning preclinical research, clinical papers, case reports, reports
on development of endoscopic tools and accessory, are welcomed. We will do our utmost to ensure prompt publication
once a paper is accepted.
We look forward to working with our colleagues in the promotion ofall aspects ofdiagnostic and therapeutic endoscopy.
Harubumi Kato, M.D.
Editor-in-Chief

				

